# Serological Assays Estimate Highly Variable SARS-CoV-2 Neutralizing Antibody Activity in Recovered COVID-19 Patients

**DOI:** 10.1128/JCM.02005-20

**Published:** 2020-11-18

**Authors:** Larry L. Luchsinger, Brett P. Ransegnola, Daniel K. Jin, Frauke Muecksch, Yiska Weisblum, Weili Bao, Parakkal Jovvian George, Marilis Rodriguez, Nancy Tricoche, Fabian Schmidt, Chengjie Gao, Shabnam Jawahar, Mouli Pal, Emily Schnall, Huan Zhang, Donna Strauss, Karina Yazdanbakhsh, Christopher D. Hillyer, Paul D. Bieniasz, Theodora Hatziioannou

**Affiliations:** a Laboratory of Stem Cell Regenerative Research, Lindsley F. Kimball Research Institute, New York Blood Center, New York, New York, USA; b Laboratory of Retrovirology, The Rockefeller University, New York, New York, USA; c Howard Hughes Medical Institute, New York, New York, USA; d Laboratory of Complement Biology, Lindsley F. Kimball Research Institute, New York Blood Center, New York, New York, USA; e Laboratory of Molecular Parasitology, Lindsley F. Kimball Research Institute, New York Blood Center, New York, New York, USA; f Laboratory of Blood-Borne Parasites, Lindsley F. Kimball Research Institute, New York Blood Center, New York, New York, USA; g Laboratory of Membrane Biology, Lindsley F. Kimball Research Institute, New York Blood Center, New York, New York, USA; h New York Blood Center Enterprises, New York, New York, USA; Cepheid

**Keywords:** COVID-19, SARS-CoV-2, antibody, immunity, immunoglobin, infection, neutralization, serology

## Abstract

The development of neutralizing antibodies (NAbs) against severe acute respiratory syndrome coronavirus 2 (SARS-CoV-2) following infection or vaccination is likely to be critical for the development of sufficient population immunity to drive cessation of the coronavirus disease of 2019 (COVID-19) pandemic. A large number of serologic tests, platforms, and methodologies are being employed to determine seroprevalence in populations to select convalescent plasma samples for therapeutic trials and to guide policies about reopening. However, the tests have substantial variations in sensitivity and specificity, and their ability to quantitatively predict levels of NAbs is unknown. We collected 370 unique donors enrolled in the New York Blood Center Convalescent Plasma Program between April and May of 2020. We measured levels of antibodies in convalescent plasma samples using commercially available SARS-CoV-2 detection tests and in-house enzyme-linked immunosorbent assays (ELISAs) and correlated serological measurements with NAb activity measured using pseudotyped virus particles, which offer the most informative assessment of antiviral activity of patient sera against viral infection. Our data show that a large proportion of convalescent plasma samples have modest antibody levels and that commercially available tests have various degrees of accuracy in predicting NAb activity. We found that the Ortho anti-SARS-CoV-2 total Ig and IgG high-throughput serological assays (HTSAs) and the Abbott SARS-CoV-2 IgG assay quantify levels of antibodies that strongly correlate with the results of NAb assays and are consistent with gold standard ELISA results. These findings provide immediate clinical relevance to serology results that can be equated to NAb activity and could serve as a valuable roadmap to guide the choice and interpretation of serological tests for SARS-CoV-2.

## Abstract (precis)

The development of neutralizing antibodies (NAbs) against severe acute respiratory syndrome coronavirus 2 (SARS-CoV-2) following infection or vaccination is likely to be critical for the development of sufficient population immunity to drive cessation of the coronavirus disease of 2019 (COVID-19) pandemic. A large number of serologic tests, platforms, and methodologies are being employed to determine seroprevalence in populations to select convalescent plasma samples for therapeutic trials and to guide policies about reopening.

## INTRODUCTION

In late 2019, a cluster of patients in Wuhan, the capital city of China’s Hubei providence, were reported to be afflicted with a severe respiratory illness of unknown origin (1, 2). Patients presented with symptoms that included high fever, pneumonia, dyspnea, and respiratory failure. The causative agent was identified as severe acute respiratory syndrome coronavirus variant 2 (SARS-CoV-2), the seventh coronavirus strain to infect humans to date (3), and the clinical syndrome was designated coronavirus disease of 2019 (COVID-19). The pathogenesis of COVID-19 is similar to that of previously documented respiratory distress syndromes caused by related coronaviruses, including the 2005 SARS-CoV and the Middle East respiratory syndrome (MERS) coronavirus (4). However, the greater transmissibility of SARS-CoV-2 has enabled a swift global spread that has resulted in substantial mortality. Detection and tracking of SARS-CoV-2 spread has been difficult. Moreover, the spectrum of symptomatology observed in SARS-CoV-2 infection is wide, ranging from asymptomatic and mild, reminiscent of numerous seasonal infections including influenza and common cold viruses, all the way to life-threatening respiratory failure that requires intensive care and invasive ventilation. Currently, increased age and comorbidities are the factors most highly predictive of severe COVID-19 disease (5).

The utility of serological tests to identify individuals who have acquired antibodies against SARS-CoV-2 is thus recognized as both an indication of the seroprevalence of SARS-CoV-2 infection and, potentially, of immunity afforded to the seropositive individual (3, 6–8). Seroconversion is determined by detection of antibodies that recognize SARS-CoV-2 antigens. Coronaviruses have 4 major structural proteins: spike (S) protein (including the S1 domain and receptor binding domain [RBD]), nucleocapsid (N) protein, membrane (M) protein, and envelope (E) protein (9). Previous studies of SARS-CoV and MERS-CoV found that the most immunogenic antigens are the S and N proteins (10), and development of serological tests for SARS-CoV-2 antibodies has focused heavily on these viral proteins.

Three major platforms of serological testing have been adopted: (i) enzyme-linked immunosorbent assays (ELISAs), (ii) high-throughput serological assays (HTSAs), and (iii) lateral flow assays (LFAs). ELISAs offer wide flexibility for research laboratories to select virtually any antigen of interest and provide highly sensitive, quantitative results. HTSAs are more suitable for clinical laboratories and offer limited antigen diversity but allow high-throughput and sensitive, semiquantitative results. LFAs also offer limited antigen diversity but function with small volumes (∼20 μl) of whole blood, plasma, or serum and allow rapid (≤15-min) results at the point of care. The clinical community will undoubtedly employ multiple SARS-CoV-2 serology platforms, but a comparative analysis across platforms has not been undertaken. Furthermore, it is currently unknown whether the detection of antibodies that bind these proteins predicts neutralizing activity or protection against infection (11).

Convalescent plasma (CP) transfusion has been recognized as a potential treatment for critically ill COVID-19 patients, and the New York Blood Center (NYBC) has led the first COVID-19 CP donation program in the United States. Using 370 unique CP donor samples deposited in our COVID-19 Research Repository (https://www.nybc.org/lindsley-f-kimball-research-institute/covid-19-research-repository/), we conducted ELISA, HSTA, and LFA assays, as well as SARS-CoV-2 pseudovirus neutralization assays. We find that CP donors have a wide range of antibody titers measured across multiple COVID-19 serological and neutralization assays. Notably, we show that some HTSAs and ELISAs predict neutralizing activity *in vitro* and may thus serve to predict antiviral activity against SARS-CoV-2 *in vivo*.

## MATERIALS AND METHODS

### Cell lines.

Huh7.5 cells were a gift from Charles Rice (12). The 293T/ACE2cl.13 cell clone was generated by transducing 293T cells (ATCC CRL-3216) with a CSIB-based lentivirus expression vector containing a cDNA encoding a catalytically inactive angiotensin-converting enzyme 2 (ACE2) mutant. Single cell clones were isolated by limiting dilution, and one clone (293T/ACE2cl.13) was used in these studies.

### Collection of CP donor information and isolation of convalescent plasma and peripheral blood mononuclear cells (PBMCs).

Disclosure of demographic information was elective at the time of donation; of the 370 CP donors analyzed, 71.1% indicated age, 95.4% indicated blood type, 95.6% indicated sex, and 55.1% indicated ethnicity. To examine the demographic characteristics within the convalescent plasma (CP) donor population, we used the 2010 U.S. Census demographic data as expected frequencies. Plasma was isolated from EDTA-anticoagulated human whole-blood samples. Samples were shipped from the NYBC Sample Management Facility overnight at 4°C and centrifuged for 5 min at 500 × *g* to facilitate plasma/cell phase separation. The resulting upper plasma layer was extracted, aliquoted to minimize future freeze-thaw cycles, and stored at −80 C. Samples were cryopreserved and stored in the NYBC COVID-19 Research Repository (https://www.nybc.org/lindsley-f-kimball-research-institute/covid-19-research-repository/).

The remaining whole-blood cellular phase was supplemented with 2 ml of 35 g/liter human serum albumin (HSA)/Dulbecco’s phosphate-buffered saline (DPBS) and diluted 1:1 with DPBS. Diluted whole blood was layered over 7 ml Ficoll-Paque premium 1.078 g/ml (GE Healthcare) and centrifuged for 20 min at 20°C and 400 × *g* without braking. Buffy coats were extracted, counted with acridine orange (AO)/propidium iodide (PI) viability stain using the Cellometer Auto2000 (Nexelom Bioscience LLC), and frozen in PBMC freezing medium (10% dimethyl sulfoxide [DMSO] in KnockOut serum replacement).

### Plasmid constructs.

The inactivated-*env* human immunodeficiency virus type 1 (HIV-1) reporter construct (pHIV-1_NL4-3_ ΔEnv-NanoLuc) was generated from a pNL4-3 infectious molecular clone (obtained through the NIH AIDS Reagent Program, Division of AIDS, NIAID, NIH, from Malcolm Martin). It contains a NanoLuc luciferase reporter gene in place of nucleotides 1 to 100 of the *nef* gene and a 940-bp deletion 3′ to the *vpu* stop codon. The vesicular stomatitis virus (VSV)-based rVSVΔG/NG/NanoLuc plasmid was generated by insertion of a cassette containing an mNeonGreen/FMDV2A/NanoLuc luciferase cDNA into rVSVΔG (Kerafast) (13) between the M and L genes. The pSARS-CoV-2 S protein expression plasmid containing a C-terminally truncated SARS-CoV-2 S protein (pSARS-CoV2_Δ19_) gene was generated by insertion of a synthetic human codon-optimized cDNA encoding SARS-CoV-2 S1 spike protein lacking the C-terminal 19 codons into pCR3.1. An ACE2 lentiviral expression vector was constructed by inserting a cDNA encoding a catalytically inactive ACE2 mutant into the lentivirus expression vector CSIB (14).

### SARS-CoV-2 pseudotype particles.

To generate (HIV/NanoLuc)-SARS-CoV-2 pseudotype particles, 293T cells were transfected with pHIV-1_NL4-3_ ΔEnv-NanoLuc reporter virus plasmid and pSARS-CoV-2-S_Δ19_ at a molar plasmid ratio of 1:0.55. The transfected cells were washed twice with PBS the following day, and at 48 h after transfection, supernatant was harvested, clarified by centrifugation, passed through a 0.22-μm filter, aliquoted, and frozen at −80°C.

To generate (VSV/NG/NanoLuc)-SARS-CoV-2 pseudotype particles, 293T cells were infected with recombinant T7-expressing vaccinia virus (vTF7-3) and transfected with rVSVΔG/NG/NanoLuc, pBS-N, pBS-P, pBS-L, and pBS-G (13). At ∼24 h posttransfection, the supernatant was collected, filtered, and used to infect 293T cells transfected with a VSV-G expression plasmid, for amplification. To prepare stocks of (VSV/NG/NanoLuc)-SARS-CoV-2 pseudotype particles, 293T cells were transfected with pSARS-CoV2_Δ19_ and infected with the VSV-G-complemented rVSVΔG/NG/NanoLuc virus. Sixteen hours later, the supernatant was collected, clarified by centrifugation, filtered, pelleted through a 20% sucrose cushion, and stored at −80°C. The viral stock was incubated with 20% I1 hybridoma supernatant (ATCC CRL-2700) for 1 h at 37°C before use.

### Neutralization assays.

To measure neutralizing antibody (NAb) activity in convalescent plasma, 5-fold serial dilutions of plasma were incubated for 1 h at 37°C in 96-well plates with an aliquot of HIV-1- or VSV-based SARS-CoV-2-pseudotyped virus containing approximately 1 × 10^3^ infectious units. Thereafter, 100 μl of the plasma/virus mixture was added to target cells (293T_Ace2_ cl.13 or Huh7.5 cells) in 96-well plates. Cells were cultured for 48 h (HIV-1 pseudotype viruses) or 16 h (VSV pseudotype viruses). Then, cells were washed twice and lysed and NanoLuc luciferase activity in lysates was measured using either the Nano-Glo luciferase assay system (Promega) and a Modulus II microplate multimode reader (Turner BioSystem) or a Glowmax Navigator luminometer (Promega). The 50% neutralizing titer (NT_50_) for plasma was determined using 4-parameter nonlinear regression in Prism 8.4 (GraphPad).

### LFAs.

Lateral flow immunoassays (LFAs) were provided by external companies. Assay cartridges contained detection bands for IgG and IgM against SARS-CoV2-specific epitopes, as well as an internal positive control. For each assay, 20 μl convalescent plasma or serum was applied to the sample pad, followed by two drops of proprietary running buffer. After 30 min, high-resolution pictures of the detection zone were taken and saved as JPEG files. All tests were performed at room temperature.

### LFA densitometry analysis.

Relative quantification of anti-SARS-CoV-2 IgG and IgM in convalescent plasma samples was performed using built-in gel analysis macros in FIJI (https://fiji.sc/). A rectangular selection covering the detection zone was analyzed using Analyze>Gels>Plot Lanes. Integrated density values were outlined manually and extracted from the resulting plot. Using Microsoft Excel, IgG and IgM values were normalized against the density of the control band.

### SARS-CoV-2-binding-antibody ELISA.

Flat-well, nickel-coated 96-well ELISA plates (Thermo Scientific) were coated with 2 μg/ml of recombinant S1 spike protein, nucleocapsid protein, or receptor binding domain (RBD) spike protein specific to SARS-CoV-2 in resuspension buffer (1% HSA in 0.01% phosphate-buffered saline with Tween 20 [PBST]) and incubated in a stationary humidified chamber overnight at 4°C. On the day of the assay, plates were blocked for 30 min with ELISA blocking buffer (3% [wt/vol] nonfat milk in PBST). Standard curves for both S1 and RBD assays were generated by using mouse anti-SARS-CoV spike protein monoclonal antibody (MAb) (clone 3A2, catalog number ABIN2452119; Antibodies-Online) as the standard. Anti-SARS-CoV-2 nucleocapsid mouse monoclonal antibody (clone 7E1B, bsm-41414M; Bioss Antibodies) was used as a standard for nucleocapsid binding assays. Monoclonal antibody standard curves and serial dilutions of convalescent donor plasma were prepared in assay buffer (1% nonfat milk in PBST) and added to blocked plates in technical duplicates for 1 h with orbital shaking at room temperature. Plates were then washed three times with PBST and incubated for 1 h with ELISA buffer containing goat anti-human IgA, IgG, IgM (heavy and light chain)-horseradish peroxidase (HRP) (catalog number ABIN100792, Antibodies-Online) and goat anti-mouse IgG2b (heavy chain)-HRP (catalog number ABIN376251; Antibodies-Online) at 1:30,000 and 1:3,000 dilutions, respectively. Plates were then washed three times, developed with Pierce TMB (3,3′,5,5′-tetramethylbenzidine) substrate for 5 min, and quenched with 3 M HCl. Absorbance readings were collected at 450 nm. Standard curves were constructed in Prism 8.4 (GraphPad Software, Inc.) using a Sigmoidal 4PL nonlinear regression (curve fit) model.

### High-throughput serology assays (HTSAs).

Convalescent donor plasma samples were barcoded and dispatched to Rhode Island Blood Center (RIBC). Samples were analyzed using the Abbott SARS-CoV-2 IgG chemiluminescent microparticle immunoassay with the Abbott Architect *i*2000SR (Abbott Core Laboratories), as well as the Vitros Immunodiagnostic Products anti-SARS-CoV-2 total Ig test and the anti-SARS-CoV-2 IgG test with the Vitros 5600 system (Ortho Clinical Diagnostics). All assays were performed by trained RIBC employees according to the respective manufacturer’s standard procedures.

### Flow cytometric analysis of PBMCs.

Cryopreserved PBMCs were thawed, filtered, and stained with a B cell or T cell antibody cocktail for 30 min in PBS. Cells were washed with PBS and analyzed with a BD LSR Fortessa 4-laser cytometer. Cytometric analysis was performed using RUO FCS Express 7 (DeNovo Software).

## RESULTS

### Characteristics of the NYC CP donor population.

**FIG 1:**
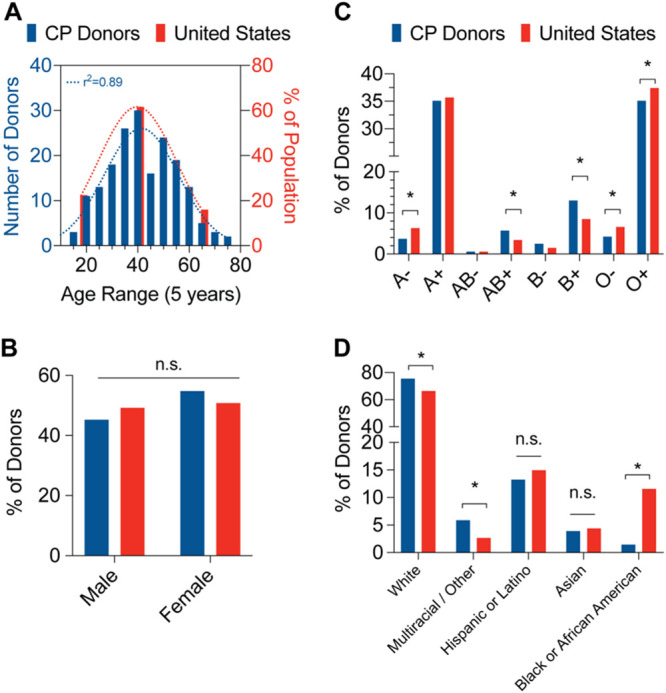
Demographics of convalescent plasma (CP) donors. (A) Distribution of convalescent plasma donor ages (left, blue bars) compared to U.S. population (right, red bars). Dotted lines represent Gaussian distribution curve fits. *n* = 263; Pearson’s correlation coefficient. (B) Distribution of convalescent plasma donor sexes (blue bars) compared to U.S. population (red bars). *n* = 354; binomial test for discrepancy versus U.S. population; n.s., not significant. (C) Distribution of convalescent plasma donor blood group antigens (blue bars) compared to U.S. population (red bars). *n* = 370; binomial test for discrepancy versus U.S. population; * *P* < 0.05. (D) Distribution of convalescent plasma donor ethnicities (blue bars) compared to U.S. population (red bars). *n* = 204; binomial test for discrepancy versus U.S. population; *, *P* < 0.05; n.s., not significant.

Serological analysis of the CP donors was performed using 370 unique samples collected between April and May of 2020 from the New York City (NYC) area. CP donors enrolled in the program were required to have tested positive for SARS-CoV-2 by PCR diagnostic tests and be symptom free for at least 2 weeks. To profile CP donors, we cross-referenced donor demographic data to the 2010 U.S. Census database (15). CP donors had a median age of 41 years (95% confidence interval [CI], 39 to 44 years; range, 17 to 75 years) and showed a Gaussian age distribution (*n* = 183, *r*^2^ = 0.89) compared to the national median age of 38.2 years in 2018 (Fig. 1A). The frequencies of male and female CP donors were 45.2% and 54.8%, respectively, not statistically different from the national average of 49.2% and 50.8% (Fig. 1B). The frequencies of ABO Rh blood group antigens were also largely consistent with the national frequencies, with slightly higher numbers of A− and O− donors and slightly lower numbers of AB+ and B+ donors than expected (Fig. 1C). Finally, CP donor ethnicities were largely consistent with the national ethnic composition, with a slightly higher number of multiracial/other donors and lower number of Black/African American donors than expected (Fig. 1D). Overall, the composition of NYC CP donors analyzed was reflective of the United States population demographic.

### Neutralizing activity among the CP donor population.

Neutralization assays measure how effectively donor plasma or serum can inhibit virus infection of target cells and are the gold standard for measuring the antiviral activity of antibodies. In the case of SARS-CoV-2, such assays require biosafety level 3 (BSL-3) facilities and highly trained personnel. To overcome this limitation and expedite testing, we employed pseudotyped-virus assays based on either human immunodeficiency virus type 1 (HIV-1) or vesicular stomatitis virus (VSV). Both viruses were engineered to lack their own envelope glycoproteins and to express a luciferase reporter gene. Complementation in *trans* with the SARS-CoV-2 spike (S) protein results in the generation of pseudotyped virus particles that are dependent on the interaction between the S protein and its receptor angiotensin-converting enzyme 2 (ACE2) for entry into cells (16). These reporter viruses were used to measure infection of human cells that were engineered to express ACE2 (HIV-S assay) or expressed endogenous ACE2 (VSV-S assay) and to determine the ability of plasma dilutions to inhibit S-dependent virus entry. The half-maximal neutralization titer (NT_50_) value, reflecting the plasma dilution at which virus infection is reduced by 50%, was calculated for each sample (Fig. S1A in the supplemental material).

**FIG 2:**
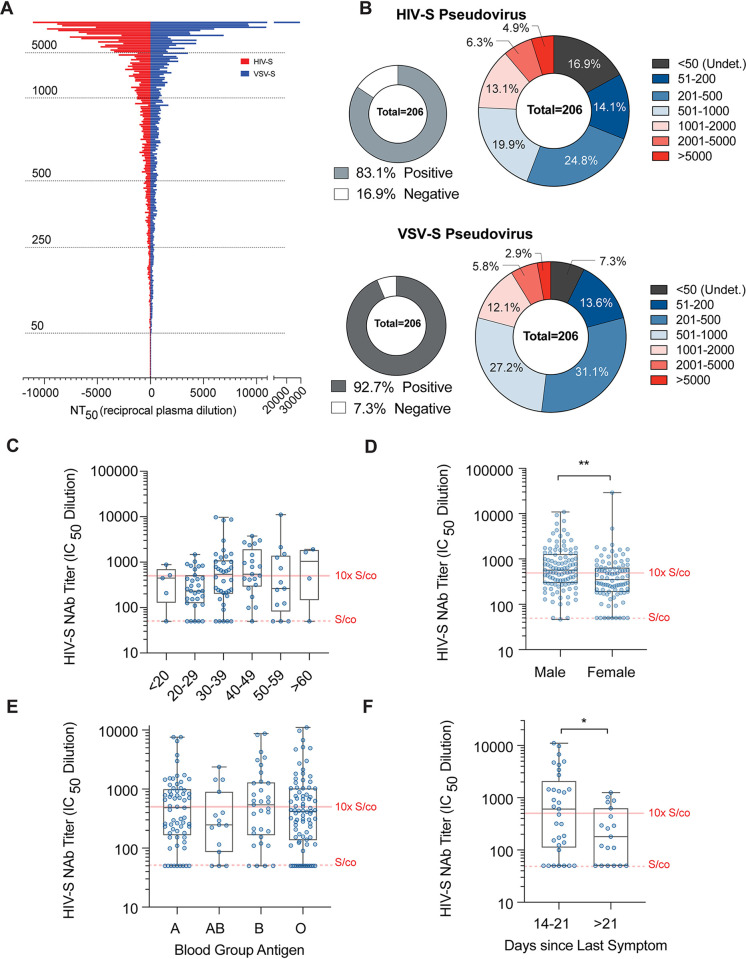
Neutralizing activity analysis of convalescent plasma (CP) donors. (A) Distribution of neutralization 50% inhibitory concentration (IC_50_) values (NT_50_, reciprocal plasma dilution) of convalescent donor plasma samples using HIV-1 (red) or VSV (blue) pseudovirus overexpressing the SARS-CoV-2 spike protein (S) (HIV-S and VSV-S, respectively). (B) Frequencies of convalescent plasma donor NT_50_ values within indicated groups using HIV-S (top) or VSV-S (bottom) pseudovirus constructs. (C) Frequency distribution of convalescent plasma HIV-S NT_50_ values versus age groups (years). *n* = 5 to 38; Kruskal-Wallis test. (D) Frequencies of convalescent plasma donor NT_50_ values versus sex. *n* = 190; Mann-Whitney test; **, *P* < 0.01. (E) Frequencies of convalescent plasma donor NT_50_ values versus blood group antigens. *n* = 15 to 82; Kruskal-Wallis test. (F) Frequencies of convalescent plasma donor NT_50_ values versus time (days) since last reported symptom. *n* = 19 to 33; Mann-Whitney *t* test; *, *P* < 0.05.

The neutralizing activities of CP donor samples were extremely variable, and the NT_50_ values obtained ranged from <50 to over 20,000. The median NT_50_ values were 390.1 (95% CI, 278.3 to 499.7) and 450.6 (95% CI, 367.7 to 538.4) for the HIV-S and VSV-S assay, respectively (Fig. 2A), and the two assays showed a high degree of correlation (Fig. S1B and C). Fresh frozen plasma (FFP) samples donated in 2019, before the SARS-CoV-2 outbreak, were used as negative controls (*n* = 10). Importantly, the NT_50_ values of all FFP samples were ≤50, which is the highest concentration of plasma used in the neutralization assays and is hence designated the signal cutoff (S/co) value. Overall, 83.1% and 92.7% of the CP donor samples had detectable neutralization activity using the HIV-S and VSV-S assay, respectively (Fig. 2B). Notably, 11.2% and 8.7% of CP donors had NT_50_ values at or greater than 2,000 (40-fold over the S/co) using the HIV-S and VSV-S assay, respectively, while 55.8% and 52% of CP donors had NT_50_ values at or less than 500 (10-fold over the S/co) (Fig. 2B). Thus, the majority of CP donors may have had relatively modest neutralizing activity and a small proportion of donors had high neutralizing activity.

NT_50_ values were not statistically different between blood groups (Fig. 2E; Fig. S1G) or age groups (Fig. 2C; Fig. S1E), and there was no linear correlation of NT_50_ values with age (Fig. S1D), in contrast to previous reports (17). However, in agreement with recent studies (18), the NT_50_ values of male CP donor samples were ∼1.7-fold higher than those of samples from female CP donors using the HIV-S and VSV-S assays (*n* = 195; *P* = 0.009 and *P* < 0.001; median difference of 217 and 197, respectively) (Fig. 2D; Fig. S1F). For CP donors where symptom dates were reported, the time between last symptom and the date of donation was calculated. Interestingly, CP donors 2 to 3 weeks postsymptoms had statistically significantly increased NT_50_ values compared to those of CP donors >3 weeks postsymptoms (*n* = 52; *P* = 0.03 and *P* = 0.04; median difference of 426 and 226, respectively) (Fig. 2F; Fig. S1H). Overall, these data suggest that CP donors possessed a wide range of neutralizing antibody (NAb) levels that were proportionately distributed across demographic categories, with the exception of a small sex-dependent effect.

### Serological test results of the CP donor population.

**FIG 3:**
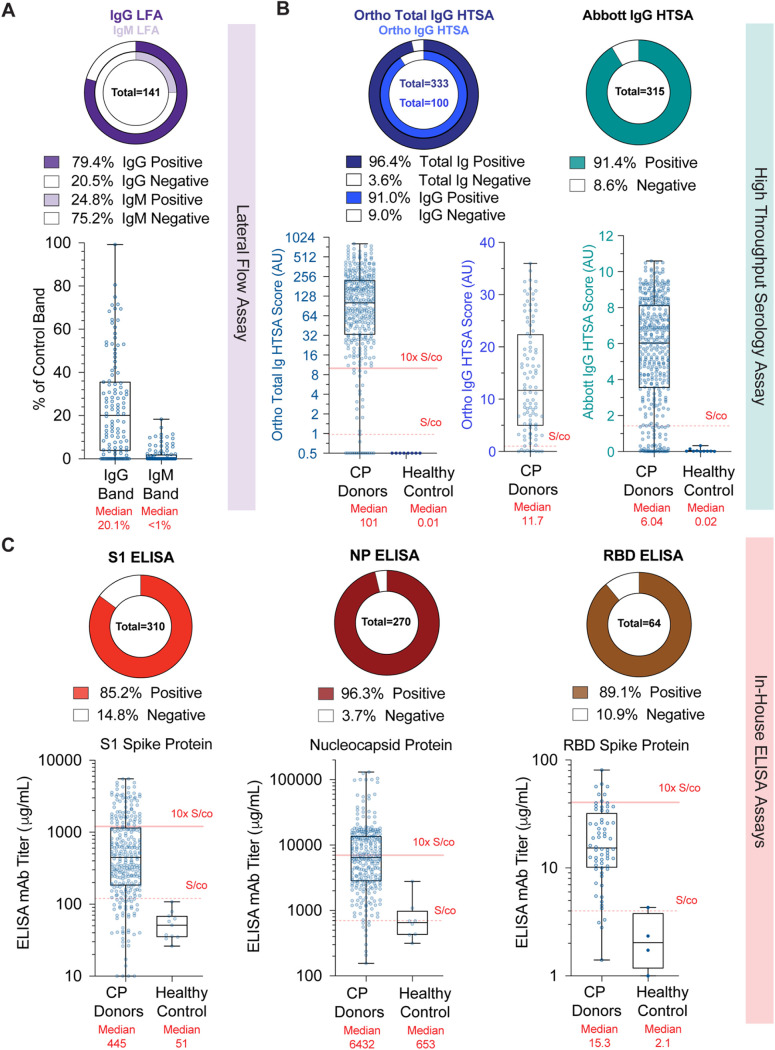
Serological analysis of convalescent plasma donors. (A) Frequencies of densitometric IgG (left) or IgM (right) results from LFA bands relative to control bands. (B) Frequencies of HTSA results using the total Ig or IgG assays derived from the Ortho Diagnostics platform (left) or Abbott IgG assay platform (right). Results from fresh frozen plasma (FFP) units collected before COVID-19 are shown as healthy controls. AU, arbitrary units. (C) Frequencies of S1 spike protein (left), nucleocapsid protein (NP) (center), and RBD spike protein (right) ELISA titer results. Titers reflect concentrations calculated using an MAb standard curve and not absolute plasma concentrations.

Multiple platforms have been deployed to detect seroconversion against SARS-CoV-2. The simplest tests are LFAs, which solubilize antibodies from whole-blood, plasma, or serum samples in an aqueous mobile phase that moves across a nitrocellulose membrane coated with anti-human IgG and/or IgM to distinguish between specific classes of immunoglobins, while a control band ensures test function. Binding of antibodies to antigen-conjugated enzyme, such as horseradish peroxidase, generates a colored band at the test lines. Analysis of 144 CP donor samples showed that only 79.4% of CP donors tested positive for SARS-CoV-2-specfic IgG antibodies and 24.8% for IgM antibodies (Fig. 3A, top). While LFAs are not designed to perform quantitatively, large discrepancies in band intensities between donors (Fig. S2A) are often presumed to indicate semiquantitative results. We performed densitometric analysis of the test bands from LFA cassettes (Fig. S2B and C) and normalized each test to the control band intensity. LFAs showed an intensity range of 0% to 99.2% for IgG bands and 0% to 18.5% for IgM bands, with median intensities of 20% for IgG and <1% for IgM (Fig. 3A, bottom). Thus, LFAs had a high degree of variation in band intensity within the CP donor population.

HTSA systems offer the advantage of performing semiquantitative seroconversion assays using clinical laboratory testing infrastructure at large scale. We performed the Ortho-Clinical Diagnostics Vitros SARS-CoV-2 total Ig assay, the Vitros SARS-CoV-2 IgG assay, and the Abbott Laboratories Architect SARS-CoV-2 IgG assay, using between 100 and 330 CP donor plasma samples. We found that 96.4% and 91.0% of CP donor samples were positive using the Ortho total Ig and IgG assays, respectively, and 91.4% were positive using the Abbott IgG assay (Fig. 3B). The median value of CP samples using the Ortho total Ig assay was 101 arbitrary units (AU) (*n* = 333; 95% CI, 78.5 to 123; S/co = 1 [range, 0 to 1,000 AU]), while that of FFP healthy controls was 0.01 AU (*n* = 8; 95% CI, 0.01 to 0.02). Similarly, the median value of CP samples using the Ortho IgG assay was 11.7 AU (*n* = 100; 95% CI, 8.3 to 16.07; S/co = 1 [range 0 to ∼30 AU]). For the Abbott assay, the median value of CP samples was 6.04 AU (*n* = 315; 95% CI, 5.48 to 6.44; S/co = 1.4 [range, 0 to ∼10 AU]), while that of FFP healthy controls was 0.02 AU (95% CI, 0.01 to 0.15). These results clearly show that HTSA platforms detected a wide variation in antibody levels in the CP donor population and offered greater dynamic range than LFA assays.

The gold standard for quantification of antigen-specific antibodies is ELISAs. Studies of antibody responses during SARS-CoV and MERS-CoV outbreaks identified the S and N proteins as the dominant antigens. Therefore, we designed three indirect ELISAs using SARS-CoV-2 recombinant, His-tagged, spike protein S1 domain (S1), spike protein RBD domain (RBD), and nucleocapsid protein (NP). We utilized monoclonal antibodies demonstrated to bind antigen in a dose-dependent manner to generate standard curves from which antibody concentrations were calculated and FFP from healthy controls to determine signal cutoffs. Thus, we report our ELISA results as monoclonal antibody (MAb) titers. These ELISAs showed that 85.2%, 89.1%, and 96.3% of CP donor samples were positive for antibodies against S1, RBD, and N antigens, respectively (Fig. 3C). Using the S1 ELISA, the median value for CP donor samples was 445 μg/ml (*n* = 285; 95% CI, 342 to 536 μg/ml; S/co = 120 μg/ml), and for FFP controls, it was 100.9 μg/ml (*n* = 10; 95% CI, 78 to 120 μg/ml). In the NP ELISA, the median value for CP donor samples was 6,432 μg/ml (*n* = 271; 95% CI, 2,811 to 13,792 μg/ml; S/co = 700 μg/ml), while in the RBD ELISA, the median value of CP donor samples was 15.6 μg/ml (*n* = 43; 95% CI, 12.55 to 25.6 μg/ml; S/co = 4 μg/ml). Notably, the range of S1- and NP-binding antibody concentrations observed in the ELISAs was extreme, constituting a 1,000-fold difference in titers within the CP donor population. Taken together, these data demonstrate that CP donors had a wide range of concentrations of antibodies specific to immunogenic SARS-CoV-2 antigens, as measured across multiple serological platforms.

### Correlation of serology tests with neutralizing activity.

**FIG 4:**
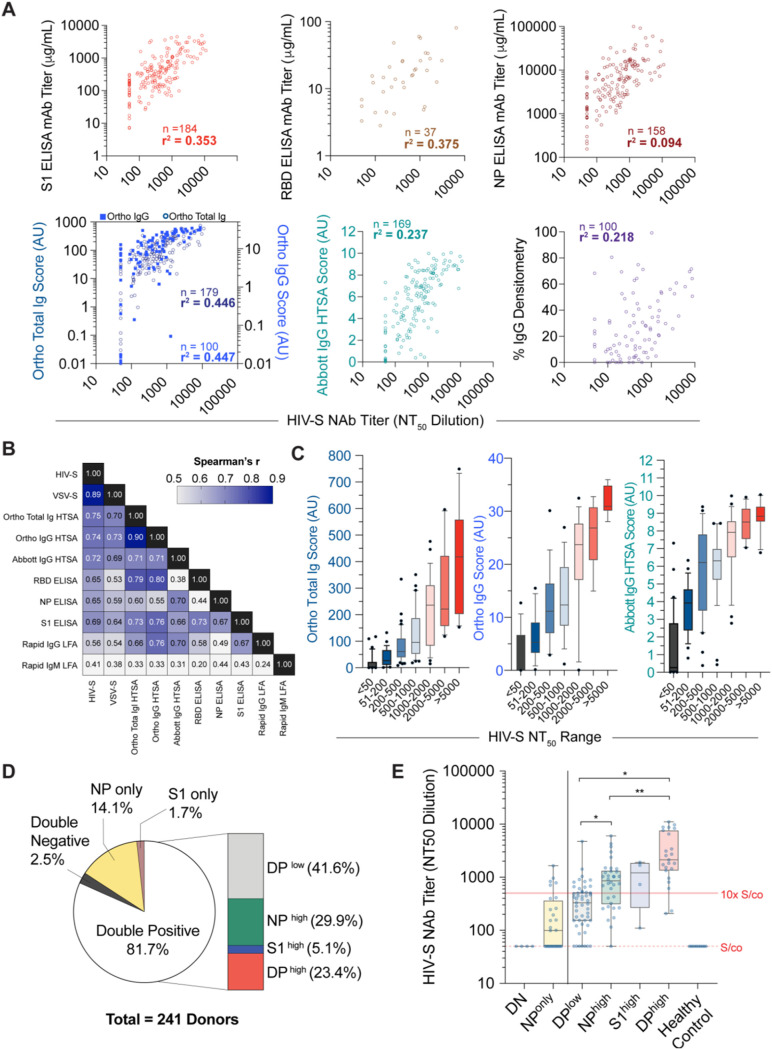
Correlation of serology assays versus neutralization activities for convalescent plasma donors. (A) Linear regression of HIV-S NT_50_ values (abscissa) versus serological assay values (ordinate). Number of samples is indicated in each graph; *r*^2^ = goodness of fit; AU, arbitrary units; NP, nucleocapsid protein. (B) Spearman correlation coefficients, *r*, of neutralization and serological assays. *n* = 137 samples; NP, nucleocapsid protein. (C) Distributions of CP donor sample HTSA scores within indicated HIV-S NT_50_ groups using Ortho total Ig (left), Ortho IgG (center), or Abbott IgG (right) assay. (D) Frequencies of convalescent donor S1 protein and nucleocapsid protein (NP) ELISA values defined in Fig. S4B in the supplemental material. *n* = 241 samples; DP, double positive. (E) Distribution of NT_50_ values corresponding to populations defined in Fig. S4B in the supplemental material. *n* = 4 to 51; Kruskall-Wallis test; *, *P* < 0.05; **, *P* < 0.01.

It is not logistically feasible to implement neutralization assays as a measurement of antiviral antibodies at a scale of the general population. While quantification of seroconversion is practiced, controlled studies that determine the relationship between quantitative SARS-CoV-2 serology test results and neutralizing activity are sparse. We examined the correlation between serology and neutralization assays in the CP donor samples (Fig. 4A; Fig. S3A and S4C). As expected, S1 ELISA titers showed a positive linear regression with NT_50_ values (*r*^2^ = 0.35), while the RBD ELISA titers showed slightly higher linearity (*r*^2^ = 0.38), commensurate with the fact that the RBD is a key target for NAbs. Conversely, NP ELISA titers showed a comparatively lower degree of linear regression with neutralization activity (*r*^2^ = 0.09). By comparison, both the Ortho HTSA total Ig assay and the IgG assay showed higher linear regression with NT_50_ values (*r*^2^ = 0.45 for both), while the Abbott HTSA IgG assay showed lower linear regression with neutralization activity (*r*^2^ = 0.24). Although Ortho HTSAs and the Abbott HTSA IgG platforms quantify antibodies against S1 and NP antigens, respectively, a linear regression of *r*^2^ = 0.33 was calculated between the Ortho total Ig HTSA and the Abbot HTSA (Fig. S3B). As expected, linear regression between the Ortho total Ig and IgG assays was strong (*r*^2^ = 0.72), since the two assays measure the same epitope. LFA IgG densitometry measurements showed the poorest correlation with neutralization activity (*r*^2^ = 0.22).

The correlations between serological results and neutralization activity were also examined using the nonparametric Spearman test that does not assume linear dependence (Fig. 4B). As expected, a high correlation between the HIV-S and VSV-S neutralization assays was obtained (*r* = 0.89). The Ortho and Abbott HTSA platforms exhibited the highest degrees of correlation with neutralization among the serology assays tested (*r* = 0.75 and *r* = 0.72, respectively, for the HIV-S assay; *r* = 0.70 and *r* = 0.69, respectively, for the VSV-S assay). The S1, RBD, and NP ELISAs also showed high degrees of correlation, particularly with the HIV-S neutralization assay (*r* = 0.69, *r* = 0.65, and *r* = 0.65, respectively), while the LFA IgG and IgM assays showed the poorest correlations (*r* = 0.56 and *r* = 0.41, respectively). Taken together, the data demonstrate that all quantitative serological assays correlate to some degree with neutralization activity. However, HTSAs and S1 ELISAs that measure anti-spike protein antigens have the highest predictive value as a surrogate for pseudovirus neutralization assays. Importantly, correlations between HTSA scores and NT_50_ values suggest presumptive ranges of neutralizing activity based on ranges of HTSA values (Fig. 4C; Fig. S4A).

While ELISAs revealed that S1 and N antibody titers correlated with each other, these titers were not always proportional among CP donor samples. To examine the coincidence of S1 and NP antibody titers and using FFP plasma samples as negative controls, we categorized S1 and N antibody titers that fell below S/co values as “negative” and titers greater than 10-fold over the S/co values as “high” (Fig. S4B). Using 241 CP donor samples that were assayed with both the S1 and N ELISAs, we found that 81% of donors were double positive (DP), while 16% of samples were single positive (14% N and 2% S1, respectively) (Fig. 4D). Only 2.5% of CP donors were double negative (DN) for S1 and NP antibodies. Within the double-positive population, we found that 23% of samples were DP^high^ (both S1 and N antibody titers were >10-fold over the S/co values), while 5% and 30% of samples were only S1^high^ or N^high^ and the remaining 42% were DP^low^ (both S1 and NP antibody titers were <10-fold higher than the S/co values). We then examined the distribution of NT_50_ values from the HIV-S neutralization assay within these populations (Fig. 4E). Notably, DN samples showed NT_50_ values at the S/co observed for FFP healthy control samples, while DP^low^ samples had relatively low NT_50_ values (median value, 327; 95% CI, 186 to 444). Importantly, the DP^high^ donors had NT_50_ values that were 7-fold higher than those of DP^low^ donors (median value, 2,130). Additionally, NT_50_ values in the N^high^ and S1^high^ groups were 2.5- and 4-fold higher than those of the DP^low^ group.

Finally, we sought to determine if the frequency of peripheral blood immune cells varied as a function of antibody titer. We stained peripheral blood mononuclear cells (PBMCs) isolated from CP donor buffy coats for classical surface markers associated with B cell or T cell populations (Fig. S5A and S5B). We examined T cell subsets, including T central memory (CD45RO^+^ CD62L^+^) and T effector memory (TEM; CD45RO^+^ CD62L^neg^), while the B cell (CD20^+^) subsets analyzed included memory B cells (CD27^+^ CD24^+^), plasmablasts (CD24^neg^ CD38^hi^ CD138^neg^), and the more mature plasma cells (CD24^neg^ CD38^hi^ CD138^+^) (Fig. S5C). We found statistically significant differences in naive CD4 and CD8 T cell populations in donors with high S1 ELISA titers compared to those with low titers. Decreases in CD24^+^ CD27^+^ memory B cells were detected in individuals with higher anti-S titers. Although the cause of this lower frequency is not known, it could raise the possibility that individuals with reduced memory B cells may develop a less robust antibody response with future infections. Although our phenotypic analysis of B and T cell compartments was limited, these data suggest that phenotypic differences in canonical B and T cell populations are insufficient to explain the large differences in antibody titers or neutralization activities observed in CP donors and warrant future studies designed to study B and T cell function from individual donors.

## DISCUSSION

### Demographic limitations of the CP donor population.

Recent studies have noted a disproportion in COVID-19 morbidity and mortality among minority communities (19). In this study, of the 370 CP samples analyzed, only 204 donors (55%) elected to identify ethnicity, representing the least reported demographic category we collected. Nevertheless, we did not observe a significant difference in NAb or serology results as a function of any demographic metric, including ethnicity. Although we showed that the CP donor samples analyzed in this study comprised a relatively normal distribution of demographic indicators, based on the U.S. census data, we acknowledge that some factors, including ethnicity, are underrepresented in this cohort and limit the interpretation of the study beyond the population aggregate. The potential explanations of this phenomenon are complex and extend beyond the scope of this study (20). The blood banking community is continuously working to recruit minority donors, who are consistently underrepresented among regular blood donors (21). Efforts to increase public participation in local blood and CP donor programs would both improve blood product diversity of transfusion products and strengthen the rigor of disease epidemiology. Thus, studies designed to characterize serological responses to COVID-19 specifically in minority groups are warranted and necessary to augment our current understanding of the pandemic.

### Seroconversion assays of the population.

Quantification of antiviral antibodies in recovered individuals is an important metric for determining population immunity conferred by exposure to SARS-CoV-2. Our study suggests that most New York City convalescent plasma donors have antibodies against SARS-CoV-2. Indeed, our data demonstrate that the HTSAs, including Ortho and Abbot assays, that have received emergency use authorization (EUA) from the FDA are well suited to quantify a wide range of antibody titers and that 91 to 96.4% of the CP population possesses detectable SARS-CoV-2 antibodies. LFAs performed less well, and individuals with low antibody titers scored weakly positive or negative in LFAs. Such outcomes could be interpreted incorrectly, thus increasing the rate of false-negative results. Ultimately, studies that accurately document SARS-CoV-2 seroprevalence in diverse populations will require highly sensitive, high-quality assays like HSTAs or ELISAs to be reliable.

### Correlation between serological assay measurements and neutralizing activity.

Since patient recovery often precedes the development of efficacious and safe therapeutics, a longstanding treatment strategy for infectious diseases is passive antibody transfer. Therefore, refining strategies to improve CP infusion efficacy both benefits the current treatment options of COVID-19 and will inform the medical community for future pandemics. Our serological analyses are consistent with previous publications that show a considerable range in antibody titers in recovered COVID-19 patients (17). However, this study provides a comprehensive analysis of the correlation of quantitative serological test values with neutralization activities. Importantly, high-dynamic-range serological assays, such as the HTSA and S1 ELISA, had significant linear correlations with neutralization activities. We show, for the first time, the extent to which three widely available SARS-CoV-2 HTSAs correlated with NAb activities, as well as to each other, providing the clinical and scientific communities with a comprehensive overview of clinical serology test performance. To this end, investigators from the Mayo Clinic’s COVID-19 Expanded Access Program (EAP) performed an exploratory analysis on the efficacy of CP as a therapeutic agent using data from over 35,000 transfusions (22). Although the study showed uncertainty as to the statistical significance of effect, the authors noted that patients transfused with high-antibody-titer CP units, quantified by the Ortho IgG assay, showed a notable reduction in the odds ratio of mortality at both 7 and 30 days after transfusion. These data support the assertion that antibody quantification of CP units using high-dynamic-range HSTA assays may further improve therapeutic options for COVID-19 and, perhaps, future pandemic responses. This knowledge will also be necessary for deriving potential serologic correlates of protection (23) and may aid in predicting immunity at the individual and population levels (18).

And yet, the levels of plasma neutralizing activity required to prevent SARS-CoV-2 reinfection are currently unknown. Anecdotal results have been reported for seasonal coronavirus experimental infection studies. For example, one study of human coronavirus 229E (HCoV-229E) found a positive correlation of preinfection antibody titers and neutralization activities with symptom clinical severity (25). In another study, 7 of 8 individuals with low neutralizing antibody titers excreted virus upon reexposure, compared to only 1 of 4 subjects with higher titers (26). However, the conclusions of these studies are not directly comparable to the current SARS-CoV-2 pandemic. As such, human epidemiological or vaccination studies are necessary to determine the minimum threshold of neutralizing activity necessary to prevent SARS-CoV-2 reinfection. Conversely, low neutralizing antibody levels have been reported to facilitate, rather than inhibit, viral entry of some coronaviruses *in vitro*, through antibody-dependent enhancement (ADE) (27–29). While ADE-dependent replication has not been demonstrated to occur in SARS-CoV, viral uptake into macrophages via antibody association with Fc receptors does induce interleukin-6 (IL-6) and tumor necrosis factor alpha (TNF-α) cytokines, which may promote inflammation and tissue damage (30). Insights gained from an accurate analysis of antibody levels and neutralization activity in SARS-CoV-2-infected individuals will help address these important questions and the corresponding health consequences.

A key biological question is, what underlies the large variation in antibody titers (neutralizing or otherwise) observed in CP donors? Numerous variables, including the effectiveness of innate immune responses, SARS-CoV-2 exposure dose, anatomical site of initial infection, and partial cross-reactive immunity conferred by prior seasonal coronavirus infection, could all impart variation on the amount and dissemination of SARS-CoV-2 antigen. Variation in the exposure of the adaptive immune system to SARS-CoV-2 antigen would, in turn, likely impact the magnitude of immune responses. Our observation that the level of antibody to N, as well as S, correlates with the S-specific neutralizing titer suggests that quantitative differences in the overall adaptive immune response to SARS-CoV-2, rather than intrinsic differences in the ability of individuals to mount neutralizing responses, at least partly explains the large variation in neutralizing capacities of CP. This notion is consistent with recent findings that all individuals examined generated very similar and potent monoclonal SARS-CoV-2 neutralizing antibodies, but at very different levels (18).

### Future utility for vaccine and CP donor strategies.

The development of efficacious vaccines against SARS-CoV-2 may be necessary for ending the COVID-19 pandemic. Clinical trials will undoubtedly include a battery of serological and neutralization assays in test subjects to assess candidate vaccine efficacies. Surrogate serology tests for neutralizing activity could help to rapidly inform as to the likely effectiveness, as well as immunogenicity, of vaccines against SARS-CoV-2. To this end, real-time analyses using scalable HTSA platforms should be effectuated while future studies are conducted to more precisely measure *in vivo* neutralization activity.

Finally, the utility of convalescent plasma in the treatment of infection has been recognized since the turn of the 20th century (31). CP transfusion is thought to be effective through passive immunization, specifically, the transfer of neutralizing antibodies from a recovered individual to another individual manifesting life-threatening symptoms (32, 33). Previously, CP therapy has been used to treat both SARS and MERS (34), and currently, it can be rapidly deployed against SARS-CoV-2 while other therapies are under development (35). Nevertheless, many questions remain regarding the optimal antibody levels necessary to treat patients at various stages of COVID-19 disease. Accurate quantification using serological assays that predict neutralization activity may improve clinical outcomes through refinement of CP unit selection for patients of varying symptomatology. In summary, we demonstrate that HTSAs and S1 ELISAs show the strongest correlations with neutralization activities and may serve to predict the degree of antiviral antibody activity present in recovered patients or vaccine recipients.

## Supplementary Material

Supplemental file 1
