# Supplementary material for: Serological Assays Estimate Highly Variable SARS-CoV-2 Neutralizing Antibody Activity in Recovered COVID-19 Patients
Source: J Clin Microbiol. 2020 Nov 18;58(12):e02005-20. doi: 10.1128/JCM.02005-20 (PMC7685895; doi:10.1128/JCM.02005-20)
Supplement: Supplemental file 1 [file JCM.02005-20-s0001.pdf]

Supplementary Material

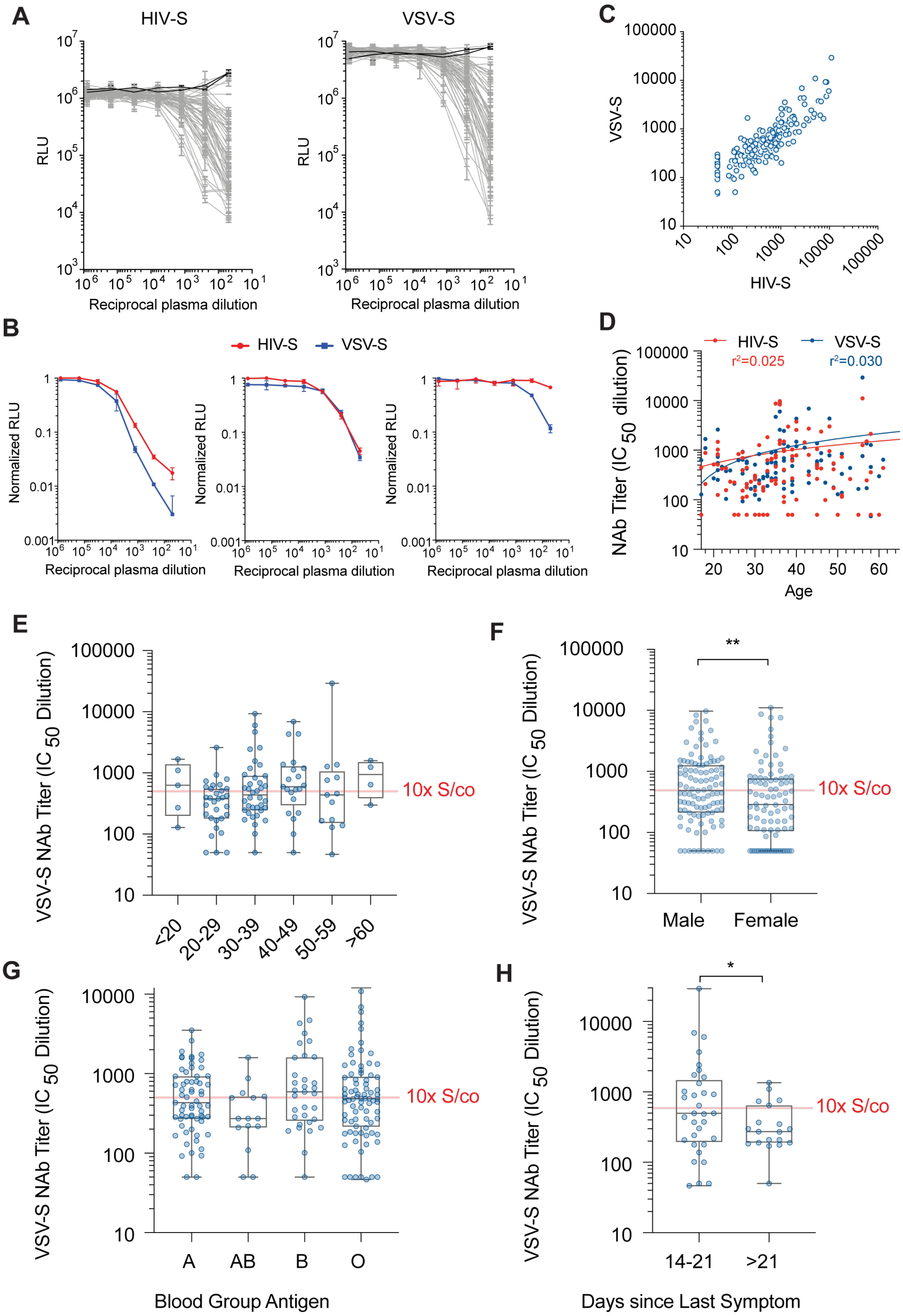

A
HIV-S
VSV-S
RLU
Reciprocal plasma dilution
C
VSV-S
HIV-S
B
HIV-S
VSV-S
Normalized RLU
Reciprocal plasma dilution
D
HIV-S
VSV-S
r2=0.025
r2=0.030
NAb Titer (IC 50 dilution)
Age
E
VSV-S NAb Titer (IC 50 Dilution)
10x S/co
<20
20-29
30-39
40-49
50-59
>60
F
**
VSV-S NAb Titer (IC 50 Dilution)
10x S/co
Male
Female
G
VSV-S NAb Titer (IC 50 Dilution)
10x S/co
A
AB
B
O
Blood Group Antigen
H
*
VSV-S NAb Titer (IC 50 Dilution)
10x S/co
14-21
>21
Days since Last Symptom

Supplementary Figure 1

A; Neutralization assay plots of serially diluted convalescent plasma (grey lines) compared to FFP controls (black lines) using HIV-S (left) or VSV-S (right) pseudovirus. Values are expressed as reciprocal plasma dilution.

B; Representative convalescent plasma donor plots classified as high (left), mid (center) and low (left) neutralization activity using HIV-S (red) or VSV-S (blue).

C; Scatterplot of HIV-S versus VSV-S NT50 values from convalescent donors.

D; Linear regression of HIV-S (red) and VSV-S (blue) NT50 values versus age. N=150, r^2 = Pearson's correlation coefficient.

E; Frequency distribution of convalescent plasma HIV-S NT50 values versus age groups. Signal to cutoff (S/co, dotted grey line) and 10x S/co (solid grey line) thresholds are indicated. n=5-38, Kruskal-Wallis test; * $p < 0.05$.

F; Frequency of convalescent plasma donor NT50 values versus sex. Signal to cutoff (S/co, dotted grey line) and 10x S/co (solid grey line) thresholds are indicated. n=190, Mann-Whitney test, ** $p < 0.01$.

G; Frequency of convalescent plasma donor NT50 values versus blood group antigen. Signal to cutoff (S/co, dotted grey line) and 10x S/co (solid grey line) thresholds are indicated. n=15-82, Kruskal-Wallis test, * $p < 0.05$.

H; Frequency of convalescent plasma donor NT50 values versus time (days) since last reported symptom. Signal to cutoff (S/co, dotted grey line) and 10x S/co (solid grey line) thresholds are indicated. n=19-33, Mann-Whitney t-test, *$p < 0.05$.

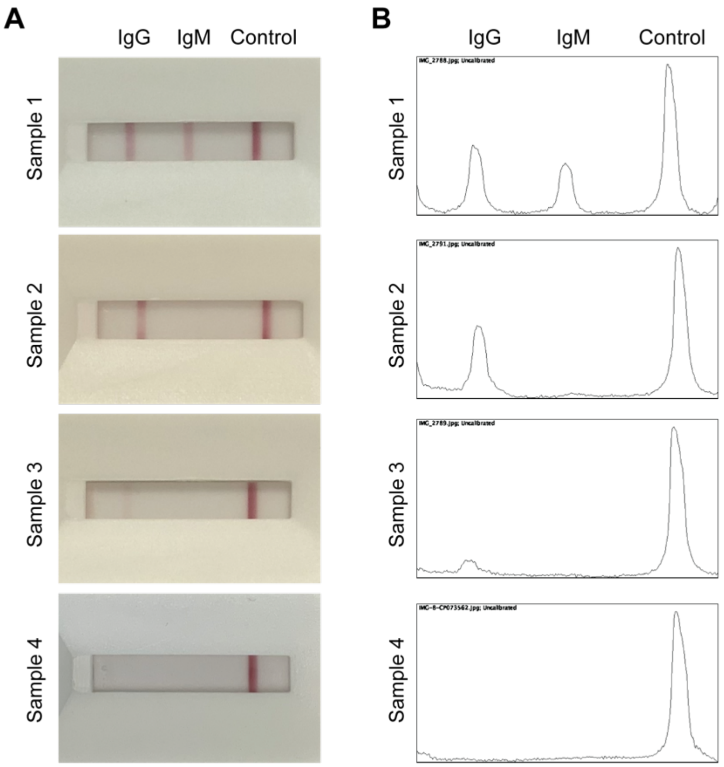

C

	Area Under Curve			Densitometry		Band Call	
Sample	Control Band	IgM Band	IgG Band	IgM / Control	IgG / Control	IgM Call	IgG Call
1	10346.3	3593.4	4940.1	34.7%	47.7%	Positive	Positive
2	10461.1	0.0	4733.0	0.0%	45.2%	Negative	Positive
3	11331.0	0.0	741.8	0.0%	6.5%	Negative	Positive
4	15609.5	0.0	0.0	0.0%	0.0%	Negative	Negative

Supplementary Figure 2

A; Representative images of LFA assays performed with convalescent plasma.

B; Densitometry plots of band intensities corresponding to images in A.

C; Transformation of data from images in A as raw data (area under curve), normalization of IgG and IgM bands to control band (Densitometry), and result assessment as “positive” or “negative”. Densitometry values ≥1% were considered positive.

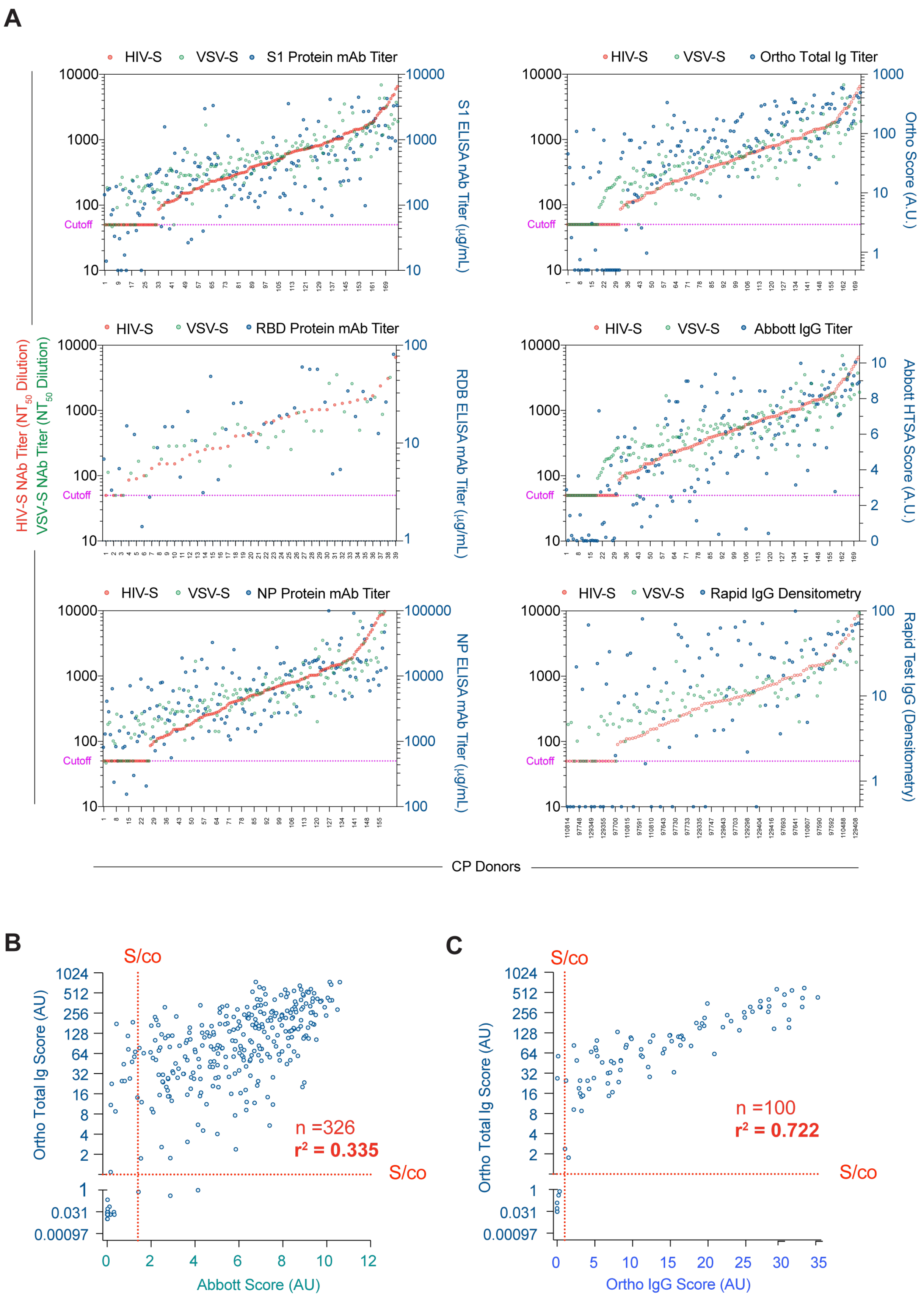
A
HIV-S
VSV-S
S1 Protein mAb Titer
S1 ELISA nAb Titer (μg/mL)
Ortho Total Ig Titer
Ortho Score (A.U.)
RBD Protein mAb Titer
RDB ELISA mAb Titer (μg/mL)
Abbott IgG Titer
Abbott HTSA Score (A.U.)
NP Protein mAb Titer
NP ELISA mAb Titer (μg/mL)
Rapid IgG Densitometry
Rapid Test IgG (Densitometry)
Cutoff
HIV-S NAb Titer (NT50 Dilution)
VSV-S NAb Titer (NT50 Dilution)
CP Donors
B
S/co
n =326
r² = 0.335
Ortho Total Ig Score (AU)
Abbott Score (AU)
C
S/co
n =100
r² = 0.722
Ortho Total Ig Score (AU)
Ortho IgG Score (AU)

Supplementary Figure 3

A; Overlaid dot plots of serology tests results (blue dots, right ordinate) aligned to rank ordered HIV-S NT50 values (red dots, left ordinate) and corresponding VIV-S NT50 values (greed dots, left ordinate). Signal-to-cutoff values are shown in dotted lines.

B; Linear regression of Ortho Total Ig HTSA values (abscissa) versus Abbott HSTA values (ordinate). N indicated in each graph, r^2 = goodness of fit.

C; Linear regression of Ortho Total Ig HTSA values (abscissa) versus Ortho IgG HSTA values (ordinate). N indicated in each graph, r^2 = goodness of fit.

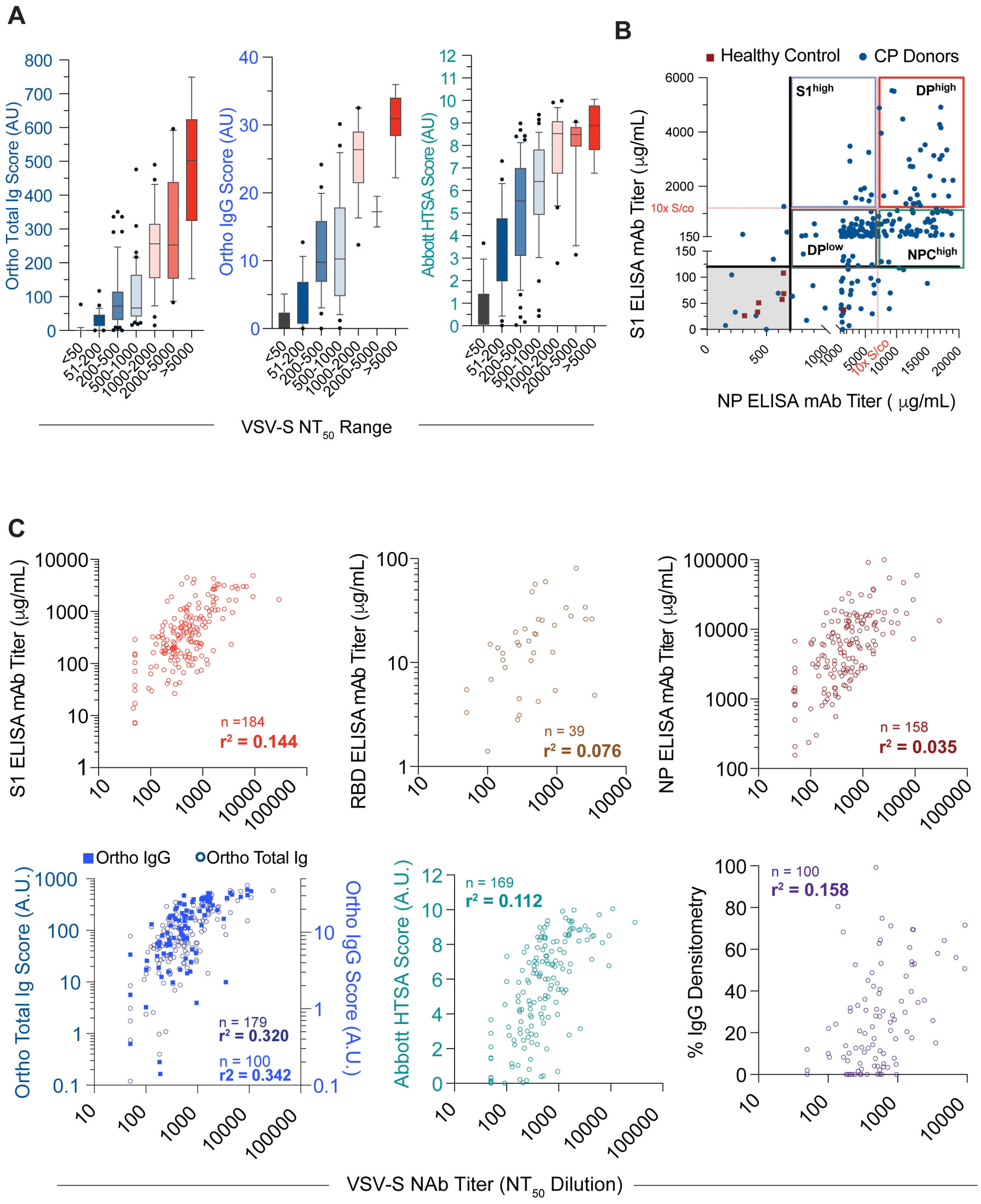

A
Ortho Total Ig Score (AU)
Ortho IgG Score (AU)
Abbott HTSA Score (AU)
<50
51-200
200-500
500-1000
1000-2000
2000-5000
>5000
VSV-S NT50 Range
B
Healthy Control
CP Donors
S1high
DPhigh
DPlow
NPChigh
10x S/co
S1 ELISA mAb Titer (µg/mL)
NP ELISA mAb Titer (µg/mL)
C
S1 ELISA mAb Titer (µg/mL)
n =184
r2 = 0.144
RBD ELISA mAb Titer (µg/mL)
n = 39
r2 = 0.076
NP ELISA mAb Titer (µg/mL)
n = 158
r2 = 0.035
Ortho IgG
Ortho Total Ig
Ortho Total Ig Score (A.U.)
Ortho IgG Score (A.U.)
n = 179
r2 = 0.320
n = 100
r2 = 0.342
Abbott HTSA Score (A.U.)
n = 169
r2 = 0.112
% IgG Densitometry
n = 100
r2 = 0.158
VSV-S NAb Titer (NT50 Dilution)

Supplementary Figure 4

A; Distribution of CP donor sample HTSA scores within indicated VSV-S NT50 groups using Ortho (left) or Abbott (right) assays.

B: Scatterplot of matched S1 spike protein versus N-protein ELISA values from convalescent donors (blue dots). FFP heathy control values are shown (red squares). Black line denotes signal/cutoff threshold and red dotted line denotes 10x signal/cutoff threshold. n=241 and 7 convalescent donor and FFP samples, respectively.

C; Linear regression of VSV-S NT50 values (abscissa) versus serological assay values (ordinate). N indicated in each graph, r^2 = goodness of fit.

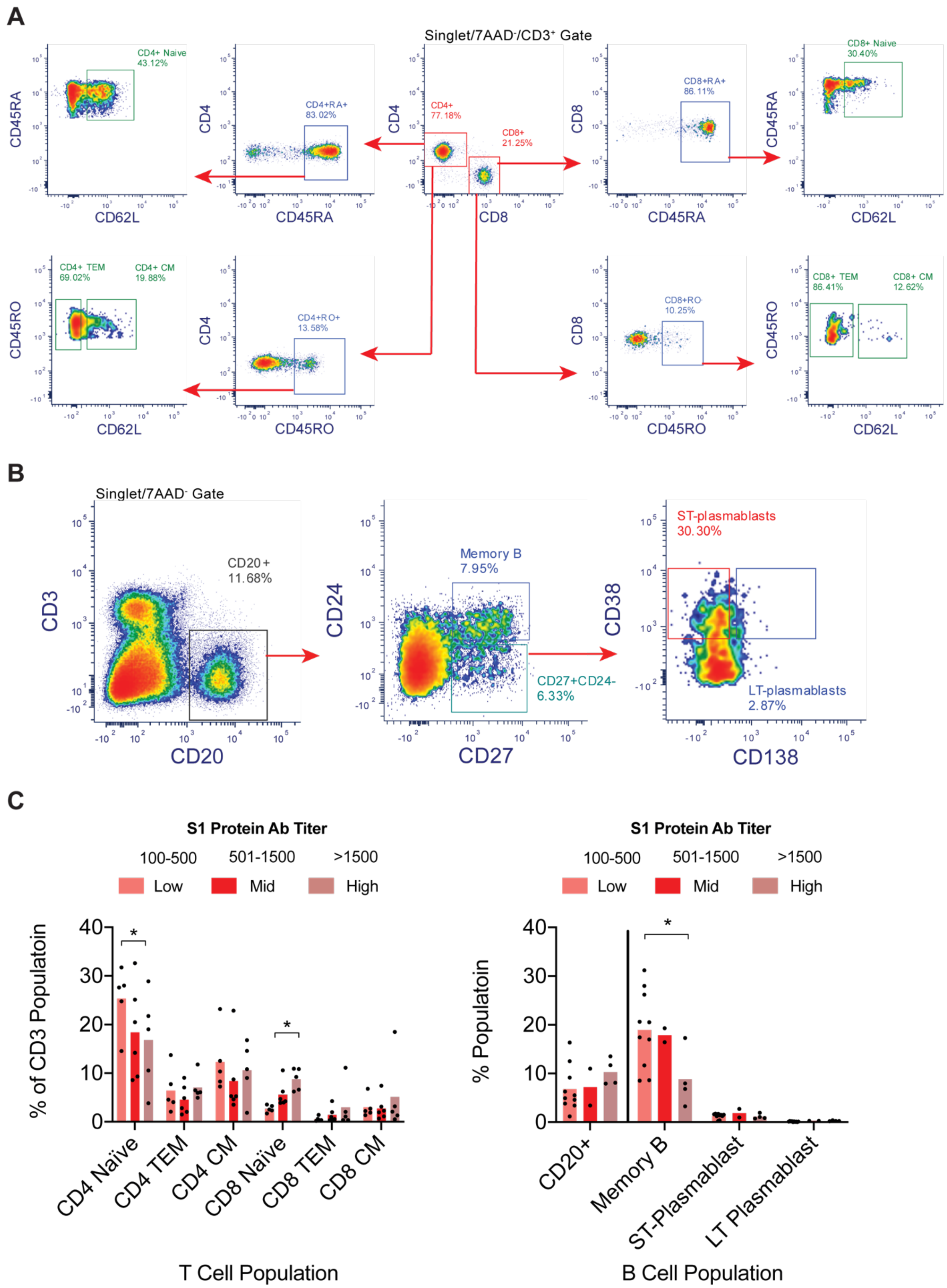
A
Singlet/7AAD-/CD3+ Gate
CD4+ Naive 43.12%
CD4+RA+ 83.02%
CD4+ 77.18%
CD8+ 21.25%
CD8+RA+ 86.11%
CD8+ Naive 30.40%
CD4+ TEM 69.02%
CD4+ CM 19.88%
CD4+RO+ 13.58%
CD8+RO 10.25%
CD8+ TEM 86.41%
CD8+ CM 12.62%
CD45RA
CD62L
CD4
CD8
CD45RO
B
Singlet/7AAD- Gate
CD20+ 11.68%
Memory B 7.95%
CD27+CD24- 6.33%
ST-plasmablasts 30.30%
LT-plasmablasts 2.87%
CD3
CD20
CD24
CD27
CD38
CD138
C
S1 Protein Ab Titer
100-500
501-1500
>1500
Low
Mid
High
% of CD3 Populatoin
CD4 Naïve
CD4 TEM
CD4 CM
CD8 Naïve
CD8 TEM
CD8 CM
T Cell Population
% Populatoin
CD20+
Memory B
ST-Plasmablast
LT Plasmablast
B Cell Population

Supplementary Figure 5

A; Gating strategy of PBMC T-cell populations from convalescent donor buffycoats. The CD4/CD8 plot (center) is sub-gated on singlet, viable, CD3+ cells.

B; Gating strategy of PBMC B-cell populations from convalescent donor buffycoats. The CD3/CD20 plot (left) is sub-gated on singlet, viable cells.

C; Quantification of PBMC T-cell (left) and B-cell (right) population frequency. Donors were grouped into low, mid and high S1 ELISA antibody titers. N=4-5 per group, two-way ANOVA, $p < 0.05$.
